# Allergic Rhinitis and Laryngeal Pathology: Real-World Evidence

**DOI:** 10.3390/healthcare9010036

**Published:** 2021-01-03

**Authors:** Yun-Ting Wang, Geng-He Chang, Yao-Hsu Yang, Chia-Yen Liu, Yao-Te Tsai, Cheng-Ming Hsu, Yi-Chan Lee, Li-Ang Lee, Pei-Rung Yang, Ming-Shao Tsai, Hsueh-Yu Li

**Affiliations:** 1 Department of Otolaryngology—Head and Neck Surgery, Chiayi Chang Gung Memorial Hospital, Chiayi 613, Taiwan; wangjulia61126@gmail.com (Y.-T.W.); genghechang@gmail.com (G.-H.C.); yaote1215@gmail.com (Y.-T.T.); scm00031@gmail.com (C.-M.H.); 2 College of Medicine, Chang Gung University, Taoyuan 333, Taiwan; r95841012@ntu.edu.tw (Y.-H.Y.); b9002063@cgmh.org.tw (Y.-C.L.); 5738@cgmh.org.tw (L.-A.L.); u9302702@cmu.edu.tw (P.-R.Y.); 3 Graduate Institute of Clinical Medical Sciences, College of Medicine, Chang Gung University, Taoyuan 333, Taiwan; 4 Health Information and Epidemiology Laboratory of Chang Gung Memorial Hospital, Chiayi 613, Taiwan; qchiayen@gmail.com; 5 Department of Traditional Chinese Medicine, Chiayi Chang Gung Memorial Hospital, Chiayi 613, Taiwan; 6 Department of Otolaryngology—Head and Neck Surgery, Keelung Chang Gung Memorial Hospital, Keelung 204, Taiwan; 7 Department of Otolaryngology—Head and Neck Surgery, Linkou Chang Gung Memorial Hospital, Taoyuan 333, Taiwan

**Keywords:** Allergic rhinitis, laryngeal pathology, unified airway, comorbidities, NHIRD

## Abstract

Allergic rhinitis (AR) is correlated with diseases including allergic laryngitis, chronic obstructive pulmonary disease (COPD), asthma, and chronic rhinosinusitis (CRS). The unified airway model suggests that inflammation can spread in both lower and upper respiratory tracts. Moreover, some voice problems—laryngeal edema, dysphonia, and vocal nodules—have been associated with AR. We examined the association between AR and laryngeal pathology. We investigated 51,618 patients with AR between 1 January 1997 and 31 December 2013, along with 206,472 patients without AR matched based on age, gender, urbanization level, and socioeconomic status at a 1:4 ratio. We followed patients up to the end of 2013 or their death. The occurrence of laryngeal pathology was the primary outcome. Individuals with AR had a 2.43 times higher risk of laryngeal pathology than the comparison cohort group (adjusted HR: 2.43, 95% CI: 2.36–2.50, *p* < 0.001). Patients diagnosed as having AR exhibited higher comorbidity rates, including of asthma, COPD, CRS, gastroesophageal reflux disease, and nasal septum deviation, than those of the comparison cohort. Our results strongly indicate that AR is an independent risk factor for laryngeal pathology. Therefore, when treating AR and voice problems, physicians should be attuned to possible laryngeal pathology.

## 1. Introduction

Allergic rhinitis (AR), which affects nearly 40% of people worldwide, is a growing public health concern. AR is commonly associated with asthma [1] under the unified airway model, which has been supported in many studies, and holds that the organs of the respiratory system, as well as the histology and pathophysiology of inflammation and hyper-reactive responses among them, are linked [1,2,3]. Thus, according to this model, inflammation can spread to the respiratory tracts, both lower and upper.

Voice problems affect up to 41 million older adults in the United States, and among younger adults, a prevalence of 6% has been noted [4,5]. Vocalization can be affected by several factors, including respiratory allergies and laryngeal disease [6,7]. In a large population-based study, the three most common laryngeal diseases, which were accompanied with voice complaints, were allergic laryngitis, vocal nodules, and vocal polyps in descending order [6].

According to the literature, fiber optic endoscopic examination has revealed that patients with AR are affected by voice symptoms and chronic edema of the vocal fold [8,9]. Moreover, a study has identified a strong link of allergic laryngitis and vocal nodules with AR [6]. Allergies may inflame the mucus from the upper to lower airway and trigger hypersecretion of mucus, leading to cough, dysphonia, and laryngeal edema [7,10]. Understanding the relationship between AR and laryngeal pathologies can help to clarify its cause and determine how to effectively treat the disease. Despite the necessity of such research, few studies have demonstrated a link between AR, laryngeal disease, and dysphonia, and no population-based literature is available on this topic. Therefore, we undertook a comprehensive real-world study by using Taiwan’s National Health Insurance (NHI) Research Database (hereafter NHIRD) to examine the relationship between AR and laryngeal pathology.

## 2. Materials and Methods

### 2.1. Design and Data

We adopted a nationwide cohort study design, employing population-based data obtained from the NHIRD. In 1995, the Taiwanese government established its NHI program, which is a universal scheme covering medical care for more than 99% of the residents of Taiwan [11]. Because of this near-universal rate of coverage, studies that analyze the data contained in the NHIRD can be regarded as national population-based studies; a wide range of information is included in the NHIRD, such as data concerning clinic visits, prescriptions, surgeries, and other procedures [12]. The NHIRD has been used in various scientific studies, and the information on diagnosis, hospitalization, and prescription use documented in the database has been proved to be of high quality. [13,14] Diagnosis codes in the NHIRD are those of the International Classification of Diseases, Ninth Revision, Clinical Modification (ICD-9-CM), and the procedure codes are those of the International Classification of Diseases, Ninth Revision, Procedure Coding System [15]. For research purposes, the National Health Research Institutes (NHRI) of Taiwan makes available the Longitudinal Health Insurance Database 2000 (LHID2000), which holds the records of one million individuals arbitrarily selected through systematic sampling from among all NHIRD registrants enrolled in the year 2000 and all claims data recorded from 1996 to 2013 [16,17]; our study data were obtained from this database. According to the NHRI, the LHID2000 sample and all NHI enrollees do not significantly differ with respect to age, sex, or socioeconomic status [18].

Approval for this study was granted by the Institutional Review Board of Chang Gung Medical Foundation (Approval #201900520B0). NHIRD data do not contain personally identifiable information, and thus, privacy of the enrolled sample was ensured. The NHI Administration and the Chang Gung Medical Foundation, through its Institutional Review Board, guaranteed that all heath and personal information would remain confidential.

### 2.2. Study Groups: With and without AR

**Figure 1:**
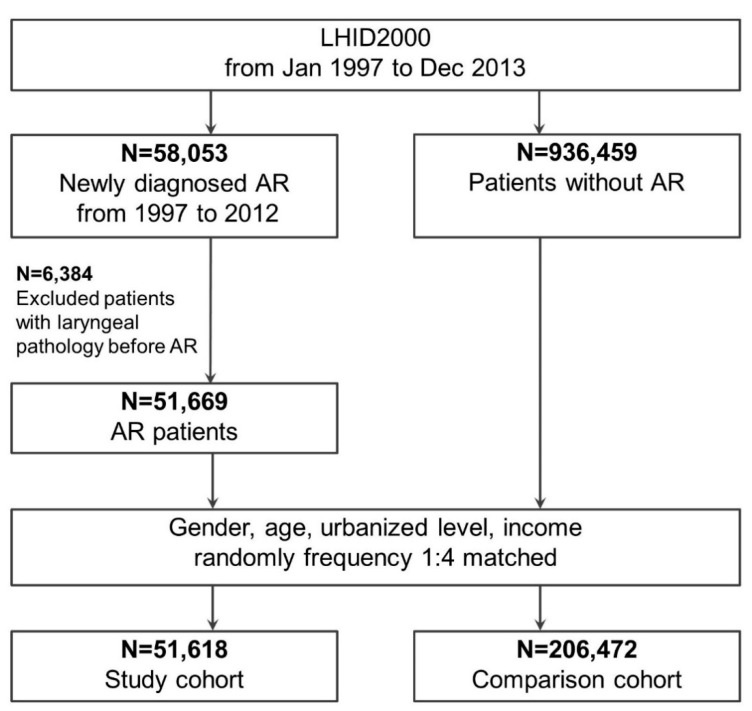
Identification and enrollment of participants. Abbreviations: AR, allergic rhinitis; LHID 2000, Longitudinal Health Insurance Database 2000.

Our analyses were conducted on 51,618 patients with AR and 206,472 comparison patients without AR; these cohorts were matched by sex, age, socioeconomic status, and urbanization level at a 1:4 ratio. The procedure for selecting participants is illustrated in Figure 1. Patients aged ≥ 0 years and who were diagnosed as having AR in the period of 1 January 1997 to 31 December 2013 were enrolled in the study cohort. Our criteria for a definite diagnosis of AR were at least one inpatient or three outpatient claims of ICD-9-CM code 477 and 477.x. Patients with laryngeal pathology (ICD-9-CM codes 478.4, 478.5, 478.6, 478.7, and 476.0) before diagnosis of AR were excluded.

For enrollment in the comparison group, we randomly selected patients without AR from the LHID2000. According to age, urbanization level, sex, and socioeconomic status, we matched these comparison patients with AR patients at a 1:4 ratio.

### 2.3. Outcome and Covariate Measurements

We followed patients until they died or the study period ended (31 December 2013). Laryngeal pathology was the primary outcome of interest (ICD-9-CM codes 478.4, vocal cord or larynx polyps; 478.5, other vocal cord diseases; 478.6, edema of larynx; 478.7, other larynx diseases not otherwise classified; 476.0, chronic laryngitis and laryngotracheitis).

The sociodemographic information of patients (age, socioeconomic status, sex, and urbanization level) was retrieved from the initial enrollment data. We searched the outpatient and inpatient claims data for comorbidities related to AR, specifically asthma [1] (ICD-9-CM code 493), chronic obstructive pulmonary disease [19] (COPD; ICD-9-CM codes 491, 492, and 496), chronic rhinosinusitis [19] (CRS; ICD-9-CM codes 473 and 473.x), gastroesophageal reflux [20] (GERD; ICD-9-CM codes 530.1, 530.10, 530.11, 530.19, and 530.81), and nasal septum deviation (NSD; ICD-9-CM codes 470) [21]. If claims data indicated that comorbidities were diagnosed one time in an inpatient scenario or three or more times in an outpatient scenario, we included them as binomial variables in statistical analysis [22].

### 2.4. Statistical Analysis

In terms of independent variables, we used a Pearson chi-squared test and the student’s t-test to analyze the categorical and sequential data, respectively, of the AR and comparison cohorts and compared their outcomes. We used the Kaplan–Meier approach to obtain the cumulative incidence rate of laryngeal pathology in both cohorts, and we applied a log-rank test for analyzing the differences between the curves of the cohorts. Moreover, Cox proportional hazard models were used to determine the adjusted hazard ratio (aHR) for laryngeal pathology. Subgroup analyses were conducted to identify potential effect modifiers and were stratified by sex, age, and comorbidities. SAS v. 9.4 (SAS Inc., Cary, NC, USA) was applied for all statistical analyses, and a two-tailed *p* < 0.05 indicated statistical significance.

## 3. Results

Our results indicated that individuals in the AR cohort had a higher likelihood of having laryngeal pathology and the comorbidities of asthma, COPD, CRS, and GERD than patients in the comparison cohort (Table 1). Moreover, the mean (SD) duration of observation was 7.4 (4.3) and 7.8 (4.3) years for the cohorts with AR and non-AR, respectively. The median time to onset of laryngeal disease in those who were diagnosed with AR was 3.2 years.

**Figure 2:**
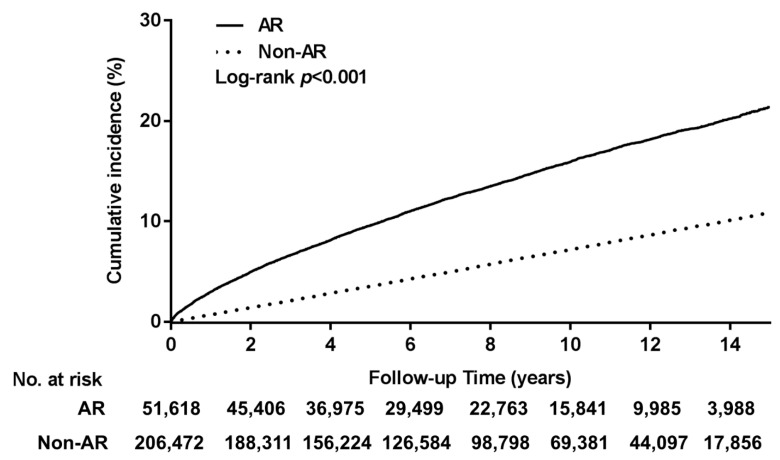
Laryngeal pathology (cumulative incidence) in individuals with and without allergic rhinitis (AR). Compared with patients without AR, those with AR had a significantly higher cumulative incidence of laryngeal pathology.

We used the Kaplan–Meier approach to identify a cumulative incidence of laryngeal pathology that was higher at the significance level in the AR cohort compared with the cohort without AR (*p* < 0.001; Figure 2). The one-, four-, and eight-year cumulative incidences of laryngeal pathology in the groups with and without AR were 3.0%, 8.1%, and 13.5% and 0.7%, 2.8%, and 5.7%, respectively.

The results of our Cox proportional hazards model of laryngeal pathology indicated that the laryngeal pathology risk in the AR cohort was 2.43 times higher compared with that in the cohort without AR (aHR: 2.43, 95% CI: 2.36–2.50, *p* < 0.001; Table 2). We performed sensitivity analyses, which entailed adding various selected covariates to the primary model; after sex, age, urbanization level, and socioeconomic status were adjusted for, AR was linked with a significantly higher risk of laryngeal pathology (Table 2). Moreover, the significant association between AR and laryngeal pathology remained in all subgroup analyses.

## 4. Discussion

This, to our knowledge, is the first nationwide study of the risk of laryngeal pathology in patients with AR. Our findings provide strong evidence that AR is a definite risk factor of laryngeal pathology. We estimated the cumulative one-, four-, and eight-year incidences of laryngeal pathology to be 3.0%, 8.1%, and 13.5%, respectively, in the cohort with AR. When comparing the cohorts with and without AR after adjustment for confounders, the aHR for laryngeal pathology was 2.43 in the AR cohort.

A study design in which a single research center is used would likely be deficient in terms of sample size and follow-up time, and thus, such a design would be inappropriate for investigating the long-term incidence of laryngeal pathology in patients with AR. By contrast, using the nationwide population-based database, namely the LHID2000, we could obtain a sufficient set of data comprising AR and laryngeal pathology cases with minimal selection bias. Because of our large sample size, our results are strong evidence that patients with AR have a relatively high occurrence of laryngeal pathology. According to the log-rank test, the incidence of laryngeal pathology in the group with AR was significantly higher compared with the group without AR. In addition, due to the relatively long mean observation time (7.72 years), we were able to identify the trends and changes in laryngeal pathology risk in both cohorts. Moreover, patients who had received a diagnosis of laryngeal pathology prior to their AR diagnosis were excluded, which helped to clarify the sequence of disease occurrence. We compared the results of the study and comparison cohorts by using matching Cox models after adjustment for comorbidities; thus, we were able to account for the influence of potential confounders. In addition, the effect of AR was consistent after subgroup analyses; we determined that, in all subgroups, laryngeal pathology risk was higher in patients with AR than in those without. Therefore, we confirmed AR to be an independent risk factor of laryngeal pathology.

AR is a commonly encountered inflammatory disease affecting the nasal mucosa. Some studies have found that AR is involved in systemic inflammation and is closely related to other inflammatory diseases and conditions, including rhinosinusitis, asthma, and allergic conjunctivitis [23]. Furthermore, the unified airway model indicates that the lower and upper respiratory airways share a common epithelium and inflammatory mediator of allergic reaction [1,2,3,24]. In studies applying the model, researchers have confirmed a higher prevalence of laryngitis in patients with AR. The correlation between AR and laryngitis may be attributable to the following mechanisms: (1) a direct inflammatory reaction in the larynx, (2) increased mucus production and its passage through the larynx, and (3) secondary edema on the vocal fold [25,26]. Because of chronic inflammation extending from the nose to the larynx, patients with AR commonly present with a cough, throat clearing, and dysphonia, all of which are highly bothersome conditions, especially among those who use their voice professionally; for instance, singers with AR have been found to have a higher prevalence of voice complaints [27]. Thus, the literature supports the existence of a correlation between AR and laryngitis, and has described possible mechanisms.

The laryngeal pathology of individuals with AR or laryngitis has only been insufficiently investigated, possibly because laryngoscopy is not a routine procedure when diagnosing AR or laryngitis. AR causes nasal congestion, nasal obstruction, and mucus hypersecretion, and triggers rhinolaryngeal reflexes, resulting in altered voice resonance, hoarseness, laryngeal edema, and dysphagia [7]. In one cohort study [6], laryngoscopy identified vocal nodules, laryngitis, Reinke’s edema, vocal polyps, and epiglottic cysts as the top five causes of voice complaints and laryngeal disease. Byeon et al. [28] reported that being middle age, being male, smoking, drinking, and having particular occupations were major risk factors for laryngeal pathology. The majority of those with laryngeal pathology can achieve improved voice quality without surgical intervention, although some patients did require voice therapy or surgical intervention [29]. Some studies have evaluated the association between AR and dysphonia, but this is the first study to document the higher risk of laryngeal pathology in individuals with AR.

We demonstrated that, compared with the non-AR cohort, the incidence rate of laryngeal pathology was higher at the significance level in the AR group. Moreover, after adjustment of some covariates, such as asthma, COPD, CRS, GERD, and NSD, the multivariate analysis revealed that AR group showed statistical significance in all subgroups. Some studies have determined that patients with AR frequently experience the comorbidities of asthma, COPD, CRS, and GERD; our results are in accordance with these findings [3,19]. When we took these comorbidities into consideration, regardless of the subgroup, patients with AR had a relatively high risk of concomitant laryngeal pathology.

Several limitations should be mentioned. First, this was a retrospective study in which we used population-based data; we lacked the original medical records to confirm diagnoses. Therefore, we used the criteria of at least three outpatient diagnoses or one inpatient diagnosis to strictly define the diseases. Second, patient symptoms, medication, smoking history, laboratory data, including serum immunoglobulin E and G, and specific allergen testing data were not available in this database; thus, we could not determine the severity of AR. Third, the diagnosis of laryngeal pathologies is based on ICD-9-CM codes, but the laryngoscopy imaging and pathology report were not available in the database; consequently, we could not diagnose laryngeal pathology in more detail. Finally, the definite mechanism responsible for the association between AR and laryngeal pathology requires further study.

## 5. Conclusions

We investigated the relationship between laryngeal pathology and AR using a national population-based database and determined AR to be an independent risk factor of laryngeal pathology. Consequently, we suggest that clinicians should be attuned to the possibility of laryngeal pathology in patients with AR. Moreover, when treating patients with AR and voice problems, a thorough laryngoscopy is suggested.

## Figures and Tables

**Table 1:** Study participants’ baseline characteristics.

Variables	Allergic Rhinitis		Non-Allergic		*p*-Value
	(N = 51,618)		(N = 206,472)		
	*n*	%	*n*	%	
Gender					1.000
Male	23,736	46.0	94,944	46.0	
Female	27,882	54.0	111,528	54.0	
Age (years)					1.000
<30	24,549	47.6	98,196	47.6	
30–60	15,926	30.9	63,704	30.9	
>60	11,143	21.6	44,572	21.6	
Urbanization level					1.000
1 (City)	17,219	33.4	68,876	33.4	
2	24,290	47.1	97,160	47.1	
3	7004	13.6	28,016	13.6	
4 (Villages)	3105	6.0	12,420	6.0	
Monthly income (NT$)					1.000
0	27,925	54.1	111,700	54.1	
1–15,840	5964	11.6	23,856	11.6	
15,841–25,000	10,564	20.5	42,256	20.5	
≥25,001	7165	13.9	28,660	13.9	
Comorbidity					
Asthma	9392	18.2	16,087	7.8	<0.001
COPD	5680	11.0	11,976	5.8	<0.001
CRS	5859	11.4	1997	1.0	<0.001
GERD	6807	13.2	12,499	6.1	<0.001
NSD	6368	12.3	1316	0.6	<0.001
Outcome	6854	13.3	12,014	5.8	<0.001

Abbreviations: NT$, New Taiwan dollar.

**Table 2:** Associations between allergic rhinitis and risk of laryngeal pathology (multivariable Cox proportional hazards regression).

Variables	Adjusted HR	95% CI		*p*-Value
Main Model *	2.43	2.36	2.50	<0.001
Additional Covariates †				
Main model + asthma	2.45	2.38	2.53	<0.001
Main model + COPD	2.43	2.36	2.50	<0.001
Main model + CRS	2.43	2.36	2.50	<0.001
Main model + GERD	2.45	2.38	2.53	<0.001
Main model + NSD	2.43	2.36	2.51	<0.001
Subgroup Effects ‡				
Gender				
Male	2.70	2.58	2.82	<0.001
Female	2.24	2.15	2.33	<0.001
Age (yrs)				
<30	1.99	1.90	2.09	<0.001
30–60	2.73	2.60	2.87	<0.001
>60	2.86	2.69	3.05	<0.001
Asthma				
Yes	2.04	1.86	2.23	<0.001
No	2.51	2.43	2.59	<0.001
GERD				
Yes	2.12	1.92	2.34	<0.001
No	2.49	2.41	2.57	<0.001
COPD				
Yes	2.53	2.28	2.80	<0.001
No	2.42	2.35	2.50	<0.001
CRS				
Yes	1.49	1.27	1.75	<0.001
No	2.47	2.39	2.54	<0.001
NSD				
Yes	1.21	1.00	1.46	0.047
No	2.47	2.39	2.55	<0.001

Abbreviations: HR, hazard ratio; CI, confidence interval. * Primary model adjusted by sex, age, urbanization, and income. † Models adjusted for primary model covariates and each additional listed comorbidity. ‡ Model adjusted for income, sex, age, and urbanization.

## Data Availability

The datasets generated or analysed in the current study can be accessed from the Taiwan National Health Insurance Research Database repository (https://nhird.nhri.org.tw/en/How_to_cite_us.html).
